# Cognitive Correlates of Different Mentalizing Abilities in Individuals with High and Low Trait Schizotypy: Findings from an Extreme-Group Design

**DOI:** 10.3389/fpsyg.2017.00922

**Published:** 2017-06-06

**Authors:** Krisztina Kocsis-Bogár, Simone Kotulla, Susanne Maier, Martin Voracek, Kristina Hennig-Fast

**Affiliations:** ^1^Department of Applied Psychology: Health, Development, Enhancement and Intervention, Faculty of Psychology, University of ViennaVienna, Austria; ^2^Department of Basic Psychological Research and Research Methods, Faculty of Psychology, University of ViennaVienna, Austria; ^3^Department of Psychiatry and Psychotherapy, Evangelisches Krankenhaus BielefeldBielefeld, Germany

**Keywords:** schizotypy, Theory of Mind (ToM), executive functions, self-agency

## Abstract

Mentalizing or Theory of Mind (ToM) deficits in schizophrenia have been studied to great extent, but studies involving samples of trait schizotypy yield ambiguous results. Executive functions like cognitive inhibition, cognitive flexibility, and agency are all prerequisites of mentalizing, and it is assumed that the impairment of these functions contributes to ToM deficits in schizophrenia. Whether these impairments influence the ToM performance of people with high trait schizotypy remains unclear. Although impaired self-agency has repeatedly been identified in people with schizotypy, its role in mentalizing is yet to be investigated. The main aim of this study was to explore whether deficits in cognitive and affective ToM can be found in high trait schizotypy, and to identify in what way these deficits are related to the positive and negative dimensions of schizotypy. The secondary aim was to examine whether these deficits correlate with executive functions. Based on the dimensional view of the schizophrenia spectrum, an extreme-group design was applied to non-clinical volunteers demonstrating high (*N* = 39) and low (*N* = 47) trait schizotypy. Affective and cognitive ToM were investigated using the Movie for Assessment of Social Cognition, a sensitive and video-based measurement. Cognitive inhibition was assessed using the Stroop Test, and cognitive flexibility was analyzed using the Trail-Making Test. Agency was measured using a computerized self-agency paradigm. Participants in the high-schizotypy group performed significantly worse in the affective ToM task (*d* = 0.79), and their overall ToM performance was significantly impaired (*d* = 0.60). No between-group differences were found with regards to cognitive ToM, executive functions, and self-agency. Cognitive flexibility correlated negatively with positive schizotypy, and contributed to a worse overall and affective ToM. Impaired cognitive inhibition contributed to undermentalizing-type errors. It was found that non-clinical participants with high trait (positive) schizotypy – especially those with slight executive-function deficits – may have difficulties in understanding the emotional state of others and consequently in functioning in social situations.

## Introduction

Impairments of Theory of Mind (ToM), or the inability to attribute intentions and mental states to others (Premack and Woodruff, 1978), is assumed to be an integral characteristic of schizophrenia (Frith, 1992), but the nature of ToM deficits and their connection to the manifestation of symptoms are still controversial. Schizophrenia is a heterogeneous concept, and the early research of Frith and colleagues (for example Corcoran et al., 1995; Pickup and Frith, 2001) showed that different symptoms of schizophrenia could be connected to different ToM deficits. Further results showed that patients with schizophrenia commit more mistakes when they attribute emotional states to others (affective ToM) compared to when they attribute thoughts or intentions to others (cognitive ToM) (Shamay-Tsoory et al., 2007). However, according to a later study conducted using the Movie for the Assessment of Social Cognition (MASC, Dziobek et al., 2006), both cognitive and affective ToM impairments are linked to schizophrenia (Montag et al., 2011). According to some neuropsychological findings ToM abilities are heterogeneous phenomena too. Results suggest that different neural structures of including the prefrontal cortex (PFC), anterior cingulate cortex, and striatum are involved in attributing cognitive, whereas networks of ventromedial and orbitofrontal cortices, the ventral anterior cingulate cortex, the amygdala and the ventral striatum in attributing affective states to others (for a review see Abu-Akel and Shamay-Tsoory, 2011).

Attributing overly simplistic mental states to others, or undermentalizing, has been connected to negative symptoms of schizophrenia (Montag et al., 2011). Contrarily, attributing overly complex mental states to others (Montag et al., 2011; Fretland et al., 2015), or overmentalizing, has been connected to positive symptoms of schizophrenia. Suspiciousness and delusions of persecution were found to be significant predictors of poor social functioning, and poor ToM performance (Hinting Test, Corcoran et al., 1995, Visual Cartoon Test, Corcoran et al., 1997) also plays a role in this relationship (Sullivan et al., 2013). However, other investigators using the Reading the Mind in the Eye Test (Baron-Cohen et al., 2001) found no connection between symptoms of schizophrenia and ToM (Kazemian et al., 2015). More research is needed to make the connection of ToM and schizophrenia symptoms clearer.

The question of the stability of ToM deficits within the schizophrenia spectrum has been debated at length. Some results suggest that ToM deficits are predominantly present in patients with acute disorganized and negative symptoms, and that remitted patients may perform just as well in false belief tasks (Pickup and Frith, 2001) and the Hinting Task (Corcoran et al., 1995) as healthy controls or patients with other psychiatric disorders. Some more recent research has revealed that ToM deficits are present in remitted patients (Wang et al., 2015), first episode patients (FEP) (Koelkebeck et al., 2010; Ho et al., 2015), ultra-high risk samples (Chung et al., 2008), and healthy siblings of patients with schizophrenia (Montag et al., 2012; Cella et al., 2015; Ho et al., 2015). Additionally, some longitudinal studies (Lysaker et al., 2011; Sullivan et al., 2014) showed that ToM deficits in FEP were still detectable 6- and 12-month follow-ups. It is not clear whether impaired ToM abilities can be also detected in healthy volunteers with high trait schizotypy, nor is it clear whether these are of similar nature to the deficits in schizophrenia and which aspect of schizotypy they are associated with. There is some evidence in support of ToM deficits in high trait schizotypy given in studies using a false-belief sequencing task (Langdon and Coltheart, 1999) and Happé’s (1994) Strange Stories Task (Pickup, 2006), but these studies do not differentiate between cognitive and affective ToM.

With regards to the types of mentalizing errors, first-grade relatives of patients with schizophrenia demonstrated higher levels of undermentalizing in cognitive ToM (Montag et al., 2012). In this respect, their performance was similar to patients with schizophrenia (Montag et al., 2011). However, no significant associations between the schizotypy dimensions and specific aspects of ToM were found in this study (Montag et al., 2012). On the one hand, ToM deficits have been associated with positive schizotypy – unusual perceptual experiences and magical thinking (Pickup, 2006; Barragan et al., 2011). On the other hand, they have also been associated with negative schizotypy – social withdrawal and anhedonia (Langdon and Coltheart, 1999). Moreover, through the use of the “Moving Shapes” (Abell et al., 2000) and Stories Task by Fletcher et al. (1995) a connection has been found between delusion-proneness and overmentalizing (Fyfe et al., 2008).

However, other studies have failed to find a connection between impaired ToM and high trait schizotypy, using TASIT (McDonald et al., 2003), a videotape-based measure (Jahshan and Sergi (2007). Similarly, Fernyhough et al. (2008) have failed to find an association between schizotypy and ToM performance using the Hinting Task and the Visual Cartoon Task. Gooding and Pflum (2011) revealed that the different results may be dependent on the variety of measures. Their participants with high positive schizotypy performed significantly worse in the Hinting Task than those with high negative or low schizotypy, whereas no between-group differences were identified using the Reading the Mind in the Eyes Test.

In order to measure social cognition, it is desirable to increase the ecological validity of the methods used and to make use of audiovisual stimuli (Dziobek, 2012). MASC is one of the few video-based measures showing complex and often ambiguous situations resembling real life scenarios (Montag et al., 2011). In some studies, MASC has been proven to be more sensitive than other non-video based measures including the Reading the Mind in the Eye Test or the Strange Stories Task, for example in differentiating individuals with Asperger syndrome from healthy controls (Dziobek et al., 2006) or detecting gender- and cortisol-dependent differences in ToM (Smeets et al., 2009). Moreover, using MASC has the advantage of presenting participants with the full complexity and dynamics of social situations. Unlike TASIT, MASC provides participants with the context of a full story, and the participants’ social interactions are interpreted within this framework (four people spending an evening together, having dinner).

The processes of attributing mental states to others and interpreting their actions require executive functions (Perner and Lang, 1999; Decety and Jackson, 2004). It is well-known that executive-function deficits are present in patients with schizophrenia (Heinrichs and Zakzanis, 1998), but it remains ambiguous as to whether these play a role in mentalizing deficits in patients. Neuropsychological findings support this proposition, regarding for example the involvement of the dorsolateral prefrontal cortex (DLPFC) in executive functions (Oldrati et al., 2016) and additionally, in the neural circuit partly responsible for cognitive ToM (Abu-Akel and Shamay-Tsoory, 2011).

Despite some contradicting results (Mazza et al., 2001; Schenkel et al., 2005; Pinkham and Penn, 2006), a large majority of studies did find a connection between poor ToM and deficits in executive functions – particularly inhibition and cognitive flexibility amongst individuals suffering from schizophrenia (for a review see Pickup, 2008). The crucial role of cognitive flexibility in the ToM performance of patient samples has been repeatedly demonstrated using several methods including a picture sequencing task (Abdel-Hamid et al., 2009) and an irony task (Champagne-Lavau et al., 2012).

Inhibition of one’s own perspective seems to be necessary to successful perspective-taking (Ruby and Decety, 2003), so it is logical to expect ToM deficits (especially of cognitive ToM) to be specifically connected to inhibition deficits in patients with schizophrenia. However, some results refer to at least a partial independence of cognitive inhibition. In a sample of schizophrenia patients, the Reading the Mind in the Eye Test found that impairments of cognitive inhibition had an effect on first-order ToM performance, but second-order ToM deficits were found to be independent of cognitive inhibition (Pentaraki et al., 2012). A case study of stroke patients with right prefrontal and temporal damage suggested that the inhibition of one’s own point of view may be a distinct neural process in inferring another person’s point of view when completing a false-beliefs task (Samson et al., 2005). These contradictions might be resolved by further results (Samson et al., 2010; Surtees et al., 2016) which suggest that level 1 perspective-taking (the ability to judge whether another person sees something) in more simple ToM tasks is possible without the involvement of cognitive functions such as inhibition, but that level 2 perspective-taking requires cognitive control.

According to the review of Giakoumaki (2012), the executive deficit caused by prefrontal dysfunction is also on the continuum similar to schizotypal traits. Some studies support the argument that high schizotypy and impaired inhibition are connected (Cimino and Haywood, 2008), particularly in cases of positive schizotypy (Louise et al., 2015). Impaired cognitive flexibility (as measured by the trail-making test or the Wisconsin Card Sorting Test) has been associated with the negative dimension of schizotypy (Louise et al., 2015; for a review see Giakoumaki, 2012). Results concerning the role of cognitive inhibition and/or cognitive flexibility as contributors to mentalizing deficits in individuals with high trait schizotypy are just as contradictory, although not as numerous as those conducted in samples with schizophrenia. It is not clear whether cognitive inhibition and flexibility have a significant effect on the differences between ToM performances in high- and low-schizotypy groups (Cella et al., 2015). It might rather be the case that some aspects of ToM deficits are mediated by general intellectual deficits (Pentaraki et al., 2012).

The knowledge of the self (Gallagher, 2000), the understanding of one’s own perspective and the ability to distinguish one’s own perspective from that of others are all prerequisites of successful mentalizing (Decety and Jackson, 2004; Bradford et al., 2015). Self-disturbances are well-known in schizophrenia (Mishara et al., 2014; Moe and Docherty, 2014), and deficits of self-perception and self-agency have been shown in the prodromal phase (Sass and Parnas, 2003) as well as in schizotypy (Platek and Gallup, 2002; Barnacz et al., 2004; Asai and Tanno, 2008). However, there is little evidence to support the connection of impaired self-agency and ToM deficits on the schizophrenia spectrum (Schimansky et al., 2010). It may be expected that agency deficits are connected to impaired ToM abilities in high trait schizotypy. According to our knowledge, this is the first study to investigate this possible association.

In light of previous inconsistent results, it seemed necessary to investigate cognitive and affective aspects of mentalizing, ToM error types, and their connections with the different dimensions of schizotypy in healthy individuals of the general population. According to our knowledge, this study is the first one ever to have done so, and the first to have analyzed the possible contribution of self-agency to ToM deficits of healthy people with high trait schizotypy. The main goal of the present study was to explore differences in ToM performance and specific ToM error types between groups with high and low trait schizotypy. The secondary aim was to find out whether participants with high and low schizotypy have different levels of cognitive flexibility, cognitive inhibition, and self-agency; or if this is not the case, to see whether differences in ToM performance and the frequency of certain error types can be further explained by impaired cognitive flexibility, cognitive inhibition and self-agency. The tertiary aim was to see whether impaired ToM performance (especially impaired cognitive ToM) is connected to either dimensions of schizotypy – positive schizotypy in particular.

## Materials and Methods

### Participants

**FIGURE 1:**
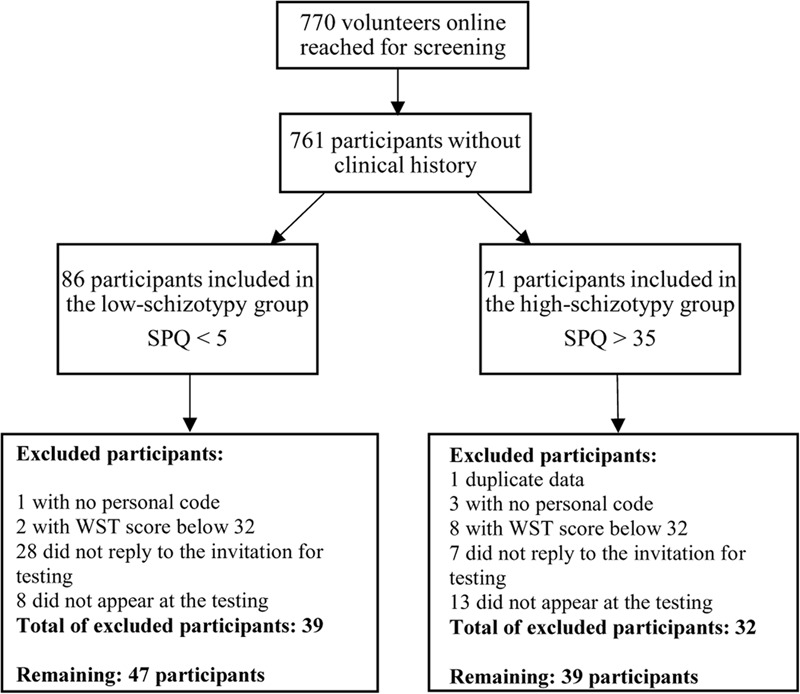
Inclusion process of participants. Abbreviations: SPQ: Schizotypal Personality Questionnaire, WST: Wortschatztest

Originally, a total of 157 healthy volunteers were reached. After the selection process described in **Figure 1**, 86 participants (72% female) were tested. Our study received approval from the local research ethics committee of the University of Vienna (nr. 00123), and is in accordance with the Declaration of Helsinki and subsequent revisions. Informed and written consent was obtained from all participants.

### Measures

#### Schizotypal Personality Questionnaire (SPQ)

Schizotypal traits were measured using the German version of SPQ (Raine, 1991; Klein et al., 1997), which contains 74 items (sample α = 0.977). Following the indications of the German translation, subscales were used to create a negative (sample α = 0.968) and a positive (sample α = 0.951) schizotypy dimension.

#### Wortschatztest

Wortschatztest (Schmidt and Metzler, 1992) contains 42 multiple-choice tasks used to measure verbal intelligence (sample α = 0.89). Each task includes four distractors and one correct target answer. Each correct answer is worth one point. The sum value of the score refers to IQ measures (*M* = 100; *SD* = 15).

#### Movie for the Assessment of Social Cognition (MASC)

Movie for the Assessment of Social Cognition (Dziobek et al., 2006) is an ecologically valid, video-based test used to evaluate subtle ToM difficulties. In this study, the original German version of the test was used (as a courtesy of Dr. Dziobek). During this test, participants watch a 15-min-long movie about four people (two men and two women) organizing and spending an evening together. Participants are then asked to answer 45 multiple-choice questions concerning the characters’ feelings (affective ToM), thoughts and intentions (cognitive ToM). Participants are given four possible answers to each question. One of these answers is correct and the others represent three types of mentalizing errors: overmentalizing (attributing overly complex mental states to others), undermentalizing (attributing overly simplistic mental states to others), and no ToM (failing to attribute mental states to others and explaining behavior based on objective factors instead).

#### Agency Manipulation Task

The computerized strange cursor paradigm of Asai and Tanno (2007) used to measure self-agency was transmitted to us courtesy of the first author. It was programmed for our study using MATLAB and Psychtoolbox Version 3 with the author’s permission. The task was presented on a screen (1920 × 1080 pixels). Participants were required to move their cursors on a black screen in the direction previously indicated by a white arrow. The movement of the cursor was visible to the participants, and randomly manipulated in 50% of the 80 trials. In manipulated trials, the movement of the cursor followed a 15°, 30°, 45° or 60° deviation. After each trial, participants were asked to judge whether the movement of their cursor was manipulated or not. This task measures the extent to which participants are able to attribute non-manipulated movements of the cursor to themselves (self-agency) and manipulated movements to an external source (the computer).

#### Stroop Test

The Bäumler version of Stroop Test (Bäumler, 1985) is designed to measure cognitive and perceptual inhibition. In nine different trials (three of each condition), participants are either instructed to read the names of colors printed in black ink aloud (read condition), or name the colors of horizontal ink blots (name condition), or read the names of colors printed in a color which does not correspond with the meaning of their name (interference condition which measures inhibition). Median reaction times as well as the number of corrected and uncorrected errors in the interference condition are obtained as measures of cognitive and perceptual inhibition.

#### Trail-Making Test (TMT)

Trail-Making Test (Reitan, 1979) is a short test used to assess both the speed at which participants processes information and their cognitive flexibility. In test A, participants are instructed to connect numbers with a constant line in ascending order. In test B, participants are instructed to connect numbers with a line in ascending order and letters in alphabetical order, constantly alternating between the two. Time is stopped for the duration of both parts of the test. We used the difference between the time taken to complete parts A and B in our statistical analysis.

### Procedure

Seven hundred and seventy individuals were reached through large community portals in Austria and screened for demographical data, schizotypy scores, and verbal IQ via an online questionnaire. Inclusion criteria for the study were: a verbal IQ score of at least 85, and age between 18 and 59 years. Exclusion criteria were: brain injury, present or past psychiatric or neurological illness, consumption of medication to treat this illness, alcohol dependence, regular drug consumption, and consumption of cannabis in the 2 weeks prior to testing.

Altogether, nine participants were excluded based on previous history of psychiatric diagnosis or treatment. The distribution of the total schizotypy score for the remaining 761 participants was recorded and analyzed. According to our analysis, schizotypy was not normally distributed in our sample (Kolmogorov–Smirnov = 0.83, *p* < 0.00), our data was positively skewed (skew = 1.023, kurtosis = 1.64), and individuals with low scores were overrepresented in our original sample. According to Preacher et al. (2005), building extreme groups from a non-normally distributed data set is an acceptable way to use an extreme-group approach.

Based both on the criteria above and their scores on Schizotypal Personality Questionnaire (Raine, 1991; Klein et al., 1997), a total of 157 participants were selected and allocated into low (lower 10%, scoring under 5) and high (upper 10%, scoring above 35) schizotypy groups. After the selection process described in **Figure 1**, 86 of these participants were tested – 47 in the low-schizotypy group and 39 in the high-schizotypy group, using computerized paradigms measuring ToM and agency and the cognitive tests measuring inhibition and flexibility.

All participants gave informed consent, and their data has been handled anonymously in both the online and personal sections of the study. All participants completing the screening were entered into a prize draw: three were selected at random and awarded 100€ each. Individuals participating for the duration of the study received a small compensation of 30€.

### Statistical Analysis

The statistical analysis of our data has been completed using the program SPSS 21. For group differences in the case of the dimensional variables *t*-test, in case of lack of homogeneity of variance Welch Test was used. Group comparisons regarding categorical variables were completed using the Chi square test, and when necessary conditions were not fulfilled, the Fisher’s Exact test was used. Three covariance analyses (ANCOVAs) were carried out in order to understand group differences of ToM; and the role of potential covariates. Covariates were selected based on previous correlational analyses in both groups where the connections between potential covariates and dependent variables were examined. Possible connections between dimensions of schizotypy and other variables were tested using Pearson’s correlation coefficient. The level of significance was set at 0.05. Bonferroni–Holm correction was used to correct for multiple testing (Holm, 1979).

## Results

The average age of the sample was 23.60 years (*SD* = 3.65). Demographics of the sample are provided in **Table 1**. Participants were allocated into two extreme groups of high (*N* = 39) and low (*N* = 47) schizotypy. These two groups did not differ significantly in mean age, gender, education level, mother tongue, and verbal intelligence. The high-schizotypy group showed significantly higher positive and negative schizotypy scores than the low-schizotypy group (**Table 2**).

**Table 1:** Demographics of the sample.

	Total*N* = 86	Low schizotypy *N* = 47	High schizotypy *N* = 39
Age	*M* = 23.60	*M* = 23.64	*M* = 23.56
	(*SD* = 3.65)	(*SD* = 3.50)	(*SD* = 3.86)
Women	72%	68.1%	76.9%
EDUCATION			
Ground school	1.2%	0%	2.6%
Secondary school	1.2%	0%	2.6%
Maturation exam	75.6%	70.2%	82.1%
Higher education	22.1%	29.8%	12.8%
MOTHER TONGUE			
German	87.2%	93.6%	79.5%
Other	12.8%	6.4%	20.5%
Verbal intelligence (Wortschatztest)	*M* = 33.83 (*SD* = 3.87)	*M* = 34.45 (*SD* = 3.15)	*M* = 33.03 (*SD* = 4.57)

**Table 2:** Coefficients of the model predicting group membership (high vs. low schizotypy) based on demographic variables.

	*B*	*SE*	*Wald*χ^2^	*Sig.*
Constant	3.33	2.92	1.31	0.25
Age	0.01	0.07	0.02	0.88
Gender	-0.46	0.54	0.72	0.40
Years of education	-0.05	0.05	1.09	0.30
German as mother tongue	1.11	0.76	2.13	0.14
Verbal intelligence (WST)	-0.10	0.07	2.05	0.15

*χ^*2*^ = 4.74, *p* = 0.79 (Hosmer and Lemeshow), *R^*2*^* = 0.09 (Cox and Snell), *R*^*2*^= 0.12 (Nagelkerke), Model χ*^*2*^* = 8.067, *p* = 0.15. Abbreviations: WST, Wortschatztest.*

According to our results, participants with high schizotypy performed significantly worse on the total of MASC [*t*(82) = 2.70, *p* = 0.008, *d* = 0.60]. They committed significantly more no-ToM-type errors (Welch *F*(1, 63.032) = 13.27, *p* < 0.001, *d* = -0.81) overall, and also committed more errors in attempting to attribute emotional states to others [Welch *F*(1, 51.007) = 16.20, *p* < 0.001, *d* = -0.90]. When compared to low-schizotypy individuals, the high-schizotypy group performed significantly worse in the affective [*t*(84) = 3.60, *p* = 0.001, *d* = 0.79], but not in the cognitive [*t*(83) = 1.90, *p* = 0.061] ToM tasks. Altogether more undermentalizing-type errors were committed by the high-schizotypy group than by the low-schizotypy group [*t*(84) = -2.57, *p* = 0.012, *d* = -0.56], and the high-schizotypy group also committed more errors in attributing thoughts and intentions (cognitive ToM) to others [Welch *F*(1, 65.815) = 5.77, *p* = 0.019, *d* = -0.53]. Participants with high- and low schizotypy did not differ significantly regarding general overmentalizing [*t*(84) = -0.86, *p* = 0.391] (**Table 3**). The high-schizotypy group performed significantly worse in the agency task when their cursor was deviated by 60^o^ [Welch *F*(1, 47.234) = 4.76, *p* = 0.034, *d* = -0.49], but did not perform worse when their cursors were deviated by smaller degrees. Therefore, they did not perform worse in the sum of correctly recognized trials. No significant between-group differences were found in cognitive flexibility or inhibition. All significant differences remained significant after Bonferroni–Holm correction (**Table 3**).

**Table 3:** Descriptive statistics and between-group differences of ToM performance, cognitive flexibility, inhibition and self-agency.

	Low-schizotypy group			High-schizotypy group					
Variable	Min.	Max.	*M* (*SD*)	Min.	Max.	*M* (*SD*)	*t*/Welch *F*^a^	*Sig.*	*d*
SCHIZOTYPY DIMENSIONS									
Positive schizotypy	0	5	1.70 (1.28)	14	43	26.92 (6.32)	40.61^a^	<0.001	5.53
Negative schizotypy	0	4	1.15 (1.04)	3	23	14.10 (5.16)	40.58^a^	<0.001	3.48
ToM PERFORMANCE									
MASC total ToM	25	44	35.91 (3.88)	21	38	33.64 (3.75)	2.70	0.008	0.60
MASC cognitive ToM	15	27	21.26 (2.62)	12	25	20.13 (2.81)	1.90	0.06	
MASC affective ToM	9	18	14.66 (1.84)	8	16	13.18 (1.96)	3.60	0.001	0.79
ToM DEFICITS									
MASC total Overmentalizing	1	13	5.00 (2.81)	1	11	5.49 (2.34)	-0.86	0.39	
MASC total Undermentalizing	0	7	2.77 (1.90)	0	11	4.00 (2.55)	-2.57	0.01	-0.60
MASC total noToM	0	4	0.98 (1.19)	0	8	2.18 (1.74)	13.27^a^	0.001	-0.81
MASC cognitive Overmentalizing	0	10	3.51 (2.30)	1	8	3.69 (1.62)	0.18^a^	0.67	
MASC cognitive Undermentalizing	0	5	1.43 (1.23)	0	7	2.23 (1.77)	5.77^a^	0.02	-0.53
MASC cognitive no ToM	0	2	0.62 (0.95)	0	5	1.03 (1.13)	-1.82	0.07	
MASC affective Overmentalizing	0	4	1.49 (1.16)	0	5	1.79 (1.22)	-1.19	0.24	
MASC affective Undermentalizing	0	4	1.34 (1.24)	0	6	1.77 (1.35)	-1.54	0.13	
MASC affective noToM	0	2	0.36 (0.57)	0	4	1.23 (1.25)	16.20^a^	<0.001	-0.90
COGNITIVE MEASURES									
Cognitive flexibility (TMT B-A)	9 s	119 s	38.04 s (26.69)	-8 s	90 s	29.62 s (18.08)	3.02^a^	0.09	
Cognitive inhibition (Stroop Int Med)	50 s	104 s	67.52 s (12.50)	42 s	97 s	65.57 s (12.39)	0.69	0.50	
Self-agency total	53	77	65.70 (6.96)	46	77	65.13 (7.42)	0.360	0.72	

*All significant differences remain significant after Bonferroni–Holm correction. Abbreviations: MASC, Movie for Assessment of Social Cognition; ToM, Theory of Mind; TMT B-A, Trail-Making Test B-A; Stroop Int Med, Stroop Interference Median.*

In order to determine whether the significant between-group differences of general ToM ability, general undermentalizing and affective ToM were caused in part by impaired executive functions or self-agency, covariance analyses were conducted. Prior to these analyses, it was checked whether possible covariates such as age, education, verbal intelligence, cognitive inhibition, cognitive flexibility, and self-agency correlated with ToM measures in the high-schizotypy group. Covariance analysis was only considered in cases of significant or (in the low-schizotypy group tendency level) correlation. Between-group differences of MASC total no ToM, and MASC affective no ToM, as well as MASC cognitive undermentalizing were not analyzed further due to a lack of homogeneity of variance [Levine’s test for MASC no ToM: *F*(1, 83) = 5.48, *p* = 0.022, MASC affective no ToM: *F*(1, 84) = 23.84, *p* < 0.001, and for MASC cognitive undermentalizing *F*(1, 84) = 6.66, *p* = 0.012]. Therefore, only the possible correlations of MASC total, MASC affective ToM, general undermentalizing and the potential covariates listed above were calculated. According to our results, age, years of education and verbal intelligence did not correlate significantly with any of the ToM measures. Consequently, their roles as possible covariates were not considered further. The median reaction time measured by Stroop Interference tables (cognitive inhibition) correlated negatively with affective ToM (*r* = -0.48^∗∗^, *p* = 0.004, *N* = 33) and general undermentalizing (*r* = -0.44^∗^, *p* = 0.01, *N* = 33). TMT B-A (cognitive flexibility) had a significant negative correlation with both general ToM (*r* = -0.34^∗^, *p* = 0.04, *N* = 37) and affective ToM (*r* = -0.37^∗^, *p* = 0.02, *N* = 39). Self-agency did not correlate significantly with any of the ToM variables listed above, and as a result it was not included in the covariance analysis (**Table 4**).

**Table 4:** Pearson’s correlations coefficients of ToM performance and age, years of education, verbal intelligence, cognitive flexibility, cognitive inhibition, and self-agency.

	High-schizotypy group			Low-schizotypy group		
	General ToM	Affective ToM	General under-mentalizing	General ToM	Affective ToM	General under-mentalizing
Age						
Pearson’s *r*	-0.10	-0.18	0.21	0.16	0.08	0.003
Significance	0.56	0.26	0.20	0.29	0.60	0.98
*N*	37	39	39	47	47	47
Years of education						
Pearson’s *r*	0.19	0.09	-0.27	0.02	-0.002	-0.25
Significance	0.27	0.58	0.10	0.91	0.99	0.09
*N*	37	39	39	47	47	47
WST						
Pearson’s *r*	0.20	0.13	0.11	0.22	0.20	-0.26
Significance	0.26	0.45	0.52	0.13	0.18	0.08
*N*	34	36	36	47	47	47
Stroop Int Med						
Pearson’s *r*	**-0.46**	-0.48^∗∗^	**0.44**^∗^	-0.09	0.01	**0.28**
Significance	**0.18**	0.004	**0.01**	0.54	0.93	**0.06**
*N*	**31**	33	**33**	46	46	**46**
TMT B-A						
Pearson’s *r*	**-0.34**^∗^	**-0.37**^∗^	0.26	**-0.30**^∗^	**-0.27**	0.03
Significance	**0.04**	**0.02**	0.11	**0.04**	**0.06**	0.83
*N*	**37**	**39**	39	**47**	**47**	47
Self-agency						
Pearson’s *r*	-0.04	0.24	-0.31	0.10	-0.001	-0.09
Significance	0.83	0.14	0.06	0.51	0.10	0.57
*N*	36	38	38	46	46	46

*Correlations referring to variables used as potential covariates in the covariance analyses are marked bold. Abbreviations: ToM, Theory of Mind; WST, verbal intelligence, TMT B-A, Trail-Making Test B-A; Stroop Int Med, Stroop Interference Median. ^∗^*p* < 0.05; ^∗∗^*p* < 0.01.*

**FIGURE 2:**
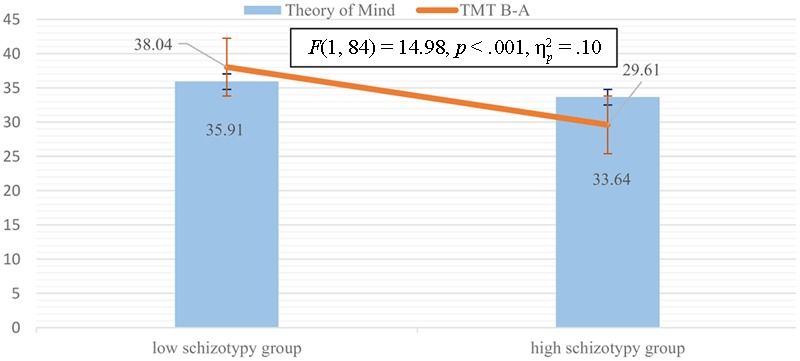
Mean difference of Theory of Mind (ToM) performance between the low- and high-schizotypy groups adjusted for the effect of cognitive flexibility.

**FIGURE 3:**
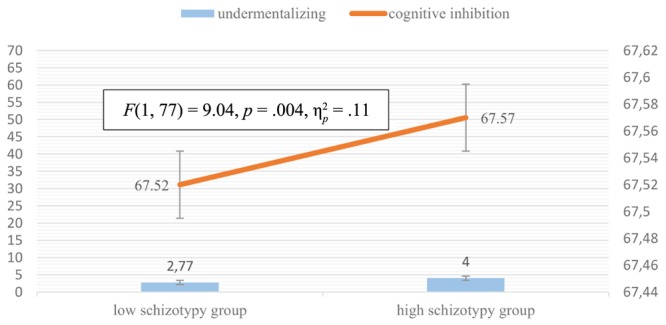
Mean difference of undermentalizing between the low- and high-schizotypy groups adjusted for the effect of cognitive inhibition.

**FIGURE 4:**
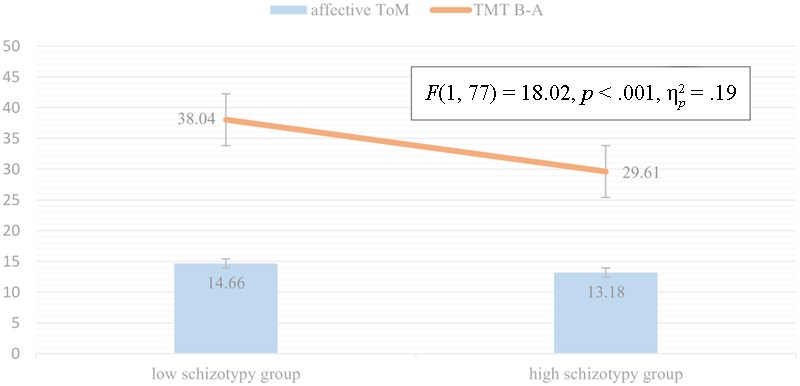
Mean difference of affective ToM adjusted for the effect of cognitive flexibility.

Three separate variance analyses were calculated based on the results of the correlation analysis detailed above. Results of ANCOVA in the case of MASC total (Levine’s Test: *p* = 0.66) showed that between-group differences in general ToM remained significant [*F*(1, 84) = 14.98, *p* < 0.001, $\eta_{p}^{2}$ = 0.15], but also showed that cognitive flexibility had an effect too [*F*(1, 84) = 10.90, *p* = 0.001, $\eta_{p}^{2}$ = 0.12] (**Figure 2**). ANCOVA in the case of general undermentalizing (Levine’s Test: *p* = 0.05) showed that differences between the high- and low-schizotypy groups remained significant even when the test was controlled for cognitive inhibition [*F*(1, 77) = 9.04, *p* = 0.004, $\eta_{p}^{2}$ = 0.106], but also showed that cognitive inhibition had a significant effect upon the results [*F*(1, 77) = 10.77, *p* = 0.002, $\eta_{p}^{2}$ = 0.124] (**Figure 3**). In the case of affective ToM, our ANCOVA (Levine’s Test: *p* = 0.82) showed significant between-group differences even after the test was controlled for cognitive flexibility [*F*(1, 77) = 18.02, *p* < 0.001, $\eta_{p}^{2}$ = 0.194], but showed that also cognitive flexibility had a significant effect [*F*(1, 77) = 6.9, *p* = 0.01, $\eta_{p}^{2}$ = 0.08] (**Figure 4**).

In order to assess whether ToM performance and certain ToM errors were connected more to the positive or the negative dimension of schizotypy, correlations between dimensions of schizotypy and the different ToM error types were examined in both groups separately. As cognitive inhibition and cognitive flexibility were not independent of general or cognitive ToM performance, these variables were also included in our analysis. Neither schizotypy dimension correlated significantly with any ToM variables measured by MASC in the high-schizotypy group, but the positive schizotypy dimension showed a significant positive correlation with TMT B-A (*r* = 0.35, *p* = 0.03, *N* = 39). This indicates a significant negative relationship between positive schizotypy and cognitive flexibility. Additionally, there was a tendency toward a positive correlation between affective no-ToM-type errors and negative schizotypy (*r* = 0.30, *p* = 0.06, *N* = 39) which did not reach significance. In the low-schizotypy group, negative schizotypy showed a significant positive correlation with cognitive (*r* = 0.33, *p* = 0.03, *N* = 47), as well as affective ToM (*r* = 0.32, *p* = 0.03, *N* = 47) and the total of ToM performance (*r* = 0.37, *p* = 0.01, *N* = 47). Positive schizotypy showed a significant correlation to affective undermentalizing (*r* = 0.34, *p* = 0.02, *N* = 47) (**Table 5**). None of the correlations remained significant after Bonferroni–Holm correction.

**Table 5:** Pearson’s correlations coefficients of schizotypy dimensions, ToM performance and cognitive measures in the high-schizotypy group.

	GeneralToM	Cogn. ToM	Aff. ToM	General over	General under	General no ToM	Cogn. over	Cogn. under	Cogn. no ToM	Aff. over	Aff. under	Aff no ToM	TMT A-B	Stroop Int Med
**HIGH-SCHIZOTYPY GROUP**														
SPQ positive														
Pearson’s *r*	-0.17	-0.17	-0.11	-0.04	0.26	-0.10	0.02	0.19	-0.07	-0.09	0.24	0.002	0.35*	0.31
Significance	0.31	0.30	0.51	0.83	0.11	0.55	0.93	0.24	0.69	0.58	0.14	0.99	0.03	0.08
*N*	39	39	39	39	39	38	39	39	38	39	39	39	39	33
SPQ negative														
Pearson’s *r*	0.12	0.24	-0.13	-0.24	-0.002	0.19	-0.27	-0.01	-0.11	-0.11	0.02	0.30	-0.23	0.19
Significance	0.49	0.15	0.43	0.13	0.99	0.25	0.10	0.93	0.50	0.49	0.93	0.06	0.17	0.30
*N*	39	39	39	39	39	38	39	39	38	39	39	39	39	33
**LOW-SCHIZOTYPY GROUP**														
SPQ positive														
Pearson’s *r*	-0.14	-0.07	-0.19	-0.08	0.20	-0.02	-0.01	-0.03	0.03	-0.16	0.34*	-0.09	0.17	-0.20
Significance	0.35	0.62	0.20	0.60	0.17	0.90	0.93	0.85	0.85	0.27	0.02	0.56	0.27	0.19
*N*	47	47	47	47	47	47	47	47	47	47	47	47	47	46
SPQ negative														
Pearson’s *r*	0.35*	0.33*	0.32*	-0.23	-0.18	-0.07	-0.23	-0.09	-0.01	-0.10	-0.19	-0.13	-0.01	0.01
Significance	0.01	0.03	0.03	0.12	0.23	0.65	0.12	0.57	0.96	0.51	0.20	0.39	0.95	0.97
*N*	47	47	47	47	47	47	47	47	47	47	47	47	47	46

*All significant correlations loose significance after Bonferroni–Holm correction. Abbreviations: SPQ, Schizotypal Personality Questionnaire; ToM, Theory of Mind; Cogn, cognitive; Aff, affective; over, overmentalizing; under, undermentalizing; TMT B-A, Trail-Making Test B-A; Stroop Int Med, Stroop Interference Median. ^∗^*p* < 0.05.*

## Discussion

According to our knowledge, this is the first study to examine the ToM performance of non-clinical volunteers with high and low trait schizotypy using MASC, and the first study to indicate that specific ToM errors can be differentiated and analyzed in relation to dimensions of schizotypy and underlying cognitive function deficits.

In line with studies of patients with schizophrenia (Fleck, 2007; Shamay-Tsoory et al., 2007; Montag et al., 2011) and healthy siblings of patients with schizophrenia (Montag et al., 2012; Cella et al., 2015) or high schizotypy (Langdon and Coltheart, 1999; Pickup, 2006), we found that people with high levels of schizotypy delivered a significantly poorer ToM performance than controls with low schizotypy. This impairment was especially pronounced in affective ToM. However, surprisingly there were no significant between-group differences with regards to cognitive ToM performance. A comparable dissociation of ToM abilities has been found in patients with schizophrenia using different methods (Shamay-Tsoory et al., 2007), but with the difference that in the cited study ToM deficits were connected to negative symptoms of schizophrenia. Our results may be partly explained by studies indicating two different but connected neuronal circuits for attributing cognitive and affective states to others (Abu-Akel and Shamay-Tsoory, 2011), but they are inconsistent with results connecting ToM deficits to the positive dimension of schizotypy (for example Pickup, 2006; Montag et al., 2011).

The high-schizotypy participants in our sample were significantly more prone to undermentalizing and no-ToM-type errors in general, cognitive undermentalizing, and affective no-ToM-type errors. From this point of view, our findings are in line with the results that Montag et al. (2012) gained from examining the relatives of schizophrenia patients. Our results are also consistent with those that Montag et al. (2011) gained from studying patients with schizophrenia; the one difference being that in the sample of Montag et al. (2011), affective undermentalizing was also higher amongst schizophrenia patients. Regarding undermentalizing and a lack of ToM, Andreou et al. (2015) reported that these two subscales were highly correlated in their sample of patients suffering from schizophrenia or borderline personality disorder (BPD), and as a result both were merged into a single undermentalizing subscale. According to their analysis, patients with schizophrenia scored significantly higher on this newly merged undermentalizing scale than both the patients with BPD and the healthy controls, whereas on the overmentalizing scale schizophrenia patients did not differ significantly from either of the other two groups (Andreou et al., 2015). Contrary to Montag et al. (2011), there were no significant between-group differences with regards to overmentalizing in the case of affective or in the case of cognitive attributions, or in summary of the two components in our sample.

No significant differences were found between the verbal intelligence, cognitive inhibition, and cognitive flexibility of the high- and low-schizotypy groups in our sample. However, our results demonstrate that the latter two of these variables have contributed significantly to the differences in ToM performance between the low- and the high-schizotypy groups. This result is especially noteworthy in this case when contrary to several studies (for reviews see Ettinger et al., 2015; Kwapil and Barrantes-Vidal, 2015), but in line with some others (Avons et al., 2003; Noguchi et al., 2008) no specific executive function deficits in connection with high schizotypy were found in our sample. Partly for this reason and partly due to their importance in the process of mentalizing, these variables are to be considered as valid covariates (Miller and Chapman, 2001).

In line with several studies showing that the deficits of cognitive flexibility contribute to an impaired ToM performance amongst schizophrenia patients (Pickup, 2008; Abdel-Hamid et al., 2009; Champagne-Lavau et al., 2012), our results indicate that cognitive flexibility exerts a significant influence over impaired overall and affective ToM performances. This result may be partly explained by the fact that cognitive flexibility is essential to the ability to take another person’s perspective (Decety and Jackson, 2004). According to our results, the impairment of this skill is especially linked to positive schizotypy. This is consistent with a previous study examining interconnections of executive functions and positive schizotypal dimensions (Louise et al., 2015), although impaired cognitive flexibility and impaired executive functions were previously more likely to be connected to the negative dimension of schizotypy (Giakoumaki, 2012). Our results are also similar to those of Cella et al. (2015), whose study of healthy siblings of schizophrenia patients recorded that cognitive flexibility deficits contribute significantly to an impaired overall ToM performance.

Additionally, we found that cognitive inhibition contributed significantly to differences in general undermentalizing. This finding is indirectly similar to that of Cella et al. (2015), who stated that a deficit in cognitive inhibition contributes to a lower ToM performance in siblings of schizophrenia patients. A possible explanation for this finding may be that an inability to inhibit one’s own simplified perception of emotions hinders the attribution of more complex emotional states to others (Decety and Jackson, 2004). This in turn leads to interpersonal difficulties including decreased interpersonal sensitivity (Miller and Lenzenweger, 2012), emotional intelligence and social functioning (Aguirre et al., 2008) amongst samples with psychometrical schizotypy. Regarding the key roles of cognitive flexibility and cognitive inhibition, our findings are in line with those of Wang et al. (2015) and indicate that these cognitive deficits – even measured independently of the ToM task itself – are strongly connected to ToM deficits in psychometrical schizotypy.

The results of our correlational analysis are to interpret with caution, because after Bonferroni–Holm correction none of them remained significant. However, there is an interesting tendency of impaired cognitive flexibility being connected to the positive and not the negative dimension of schizotypy in our high-schizotypy group. This contrasts Montag et al. (2011), who found that the ToM deficits of schizophrenia patients connected to their negative symptoms. At the same time, it is partly in line with Barragan et al. (2011), who found that ToM deficits were connected to positive and not negative schizotypy in their sample of adolescents with psychotic-like experiences. Based on the characteristics of their sample, they interpreted this finding as a developmental impairment (Barragan et al., 2011). Here it is important to mention that the German version of SPQ contains only positive and negative dimensions, and does not distinguish a disorganized dimension. Consequently, items originally intended to measure disorganized schizotypy are included in the positive and negative schizotypy subscales. It may be interesting for further research to use either a different version or a different measure to investigate whether there is a connection between the independent disorganized schizotypy dimension and cognitive flexibility. Further positive tendency-level correlations were found between negative schizotypy and different aspects of ToM, as well as positive schizotypy and affective undermentalizing in the low-schizotypy group. The correlation of positive schizotypy and affective undermentalizing is especially interesting as regards to previous results showing high affective undermentalizing amongst schizophrenia patients (Montag et al., 2011). The fact that this trend could be found even in a sample with very low schizotypy scores means further support for the dimensional view of the schizophrenia spectrum. The positive correlations between the negative schizotypy dimension and cognitive as well as affective ToM performance in the low-schizotypy group are surprising and hard to interpret. At the same time, one must be cautious at interpreting correlations of the low-schizotypy group. It has to be taken into account that this group covers a very small range of schizotypy scores. Any results need to be checked again in future studies.

Contrary to our expectations, no significant correlation could be found between ToM performance or ToM error types and the dimensions of schizotypy. There was a tendency toward a correlation between affective no-ToM-type mistakes and negative schizotypy which did not reach significance. Similar interrelatedness between no ToM and the negative dimension has been found in samples with schizophrenia, high schizotypy, and healthy siblings of schizophrenia patients (Barragan et al., 2011; Montag et al., 2011, 2012).

With regards to self-agency, people with high schizotypy only performed significantly worse than controls in the condition of rotation with 60°. This result is in line with other results obtained by studies analyzing samples with high schizotypy (Asai and Tanno, 2007, 2008) or schizophrenia (Sass and Parnas, 2003). This difference can be understood as a tendency of people in the schizophrenia-schizotypy spectrum to interpret strongly manipulated movements erroneously as their own. Deficits of self-agency did not seem to contribute to between-group differences in attributing mental states to others. In line with the findings of Schimansky et al. (2010), the ability to recognize non-manipulated movements as one’s own and manipulated movements as the movements of others seemed to be independent of an impaired perception of the mental states of others within the schizotypy group. One possible explanation for this could be the lack of self-relevance displayed by subjects undergoing the MASC. It would be interesting for future research to compare measures of ToM where participants are merely passive viewers of experimental situations to experimental situations in which individuals actively participate.

Our use of the extreme-group approach was supported by circumstances such as the time-consuming nature of some of our measures and the exploratory nature of our study in a field which mainly yields contradictory results. At the same time, the use of the extreme-group approach models comparisons of patients and healthy controls or different patient groups. This way our results are comparable to the ones of clinical studies with schizophrenia patients.

Our findings are limited not only by our relatively small sample size, but also by the fact that we used additional measures of executive functions and self-agency which were not integrated in the ToM task. As another limitation the gender proportion of our sample must be mentioned. Seventy-two percent of our participants were women which is not typical of the samples with schizophrenia where men tend to be slightly overrepresented [male:female index approximating 1.4:1 (Abel et al., 2010); men having a 1.15-fold greater risk (95% CI 1.00–1.31) than women (van der Werf et al., 2014)].

With regards to the whole of the schizotypy spectrum, our results have implications for over 10% of the population (Cohen et al., 2015). According to our study, which is partly based on underlying slight cognitive deficits, there might be some obvious deficits of social cognition present even in young, relatively highly educated individuals with high trait schizotypy, even if they do not experience psychiatric, neurological symptoms and have never previously received psychiatric treatment. Similar deficits of social cognition are connected with a lower self-rated quality of life in psychiatry patients (Brosey and Woodward, 2015), and have been found to be related to the negative dimension of schizotypy in particular (Morrison et al., 2013). It has been argued that the possible over-activity and consequent structural asymmetry of the TPJ might compensate for a lower performance of the social cognition in high trait schizotypy (Cohen et al., 2015). However, these subclinical deficits can be addressed at a young age through the development of social skills. These deficits can also be combatted using early preventive training programs (Maag, 2006) focusing on the attribution of emotional states or even possibly on the improvement of executive functions, similar to the cognitive remediation of schizophrenia (Revell et al., 2015). For further research, it is necessary to understand how different aspects of ToM deficits have psychological consequences: how they contribute to the difficulties an individual faces in adjusting to their social environment, an individual’s quality of life, and the alternative coping strategies used by people with high trait schizotypy.

## Author Contributions

KK-B and SM conceived and designed the study in consultation with KH-F. KK-B, SK, and SM conducted the study and analyzed the data with assistance and contributions from MV and KH-F. KK-B drafted the manuscript with contributions from SK, SM, MV, and KH-F. All authors read and approved the final manuscript.

## Conflict of Interest Statement

The authors declare that the research was conducted in the absence of any commercial or financial relationships that could be construed as a potential conflict of interest.
